# Diagnostic Challenge of Hepatopulmonary Syndrome in a Patient with Coexisting Structural Heart Disease

**DOI:** 10.1155/2011/386709

**Published:** 2011-09-18

**Authors:** Jorge M. Hurtado-Cordovi, Seth Lipka, Jaspreet Singh, Ghulamullah Shahzad, Paul Mustacchia

**Affiliations:** Division of Gastroenterology, Department of Medicine, Nassau University Medical Center, 2201 Hempstead Turnpike, East Meadow, NY 11554, USA

## Abstract

Hepatopulmonary syndrome (HPS) is a severe complication seen in advance liver disease. Its prevalence among cirrhotic patients varies from 4–47 percent. HPS exact pathogenesis remains unknown. Patient presents with signs/symptoms of chronic liver disease, and dypsnea of variable severity. Our patient is a 62 years old white male with a known history of chronic hepatitis C, cirrhosis, ascites, and hypothyroidism who presented to GI/liver clinic complaining of 1 episode BRBPR, and exacerbating dypsnea associated with nausea and few episodes of non-bloody vomit. Physical exam showed, icterus, jaundice, few small spider angiomas on the chest, decrease breath sounds bilateral right more than left, and mild tachycardic. Abdominal exam revealed mid-line scar, moderated size ventral hernia, distention, diffused tenderness, and dullness to percussion. Laboratory result: CBC 5.2/13.2/37.6/83, LFTs 83/217/125/5.2/4.7/7.4, Pt 22.6 INR 1.9 PTT35.4. CT scan showed liver cirrhosis, abdominal varices, and moderated ascites collection around ventral hernia. Calculated A-a gradient was 49.5. Echocardiography revealed patent foramen ovale (PFO) with predominant left to right shunt. In our case, existence of paten foramen ovale (PFO) and atelectasis precludes definitive diagnosis of HPS. Presence of cardiopulmonary shunt could be partially responsible for the patient's dypsnea exacerbation.

## 1. Introduction

Hepatopulmonary syndrome (HPS) is a severe complication seen in advance liver disease. Its prevalence among cirrhotic patients varies from 4–47 percent [1, 2]. HPS exact pathogenesis remains unknown. Excessive productions of nitric oxide by the splanchnic circulation, inducing pulmonary vasodilation and promoting angiogenesis, as well as defective clearing of bacterial endotoxins and vasodilators by the failing liver are thought to play a role in the pathophysiology of HPS. Patient presents with signs/symptoms of chronic liver disease (clubbing, ascites, spider nevi, etc.), and dyspnea of variable severity; other symptoms such as platypnea and orthodeoxia are highly specific. This entity is a diagnosis of exclusion since primary pulmonary disease and structural heart disease must be ruled out first. The diagnostic criterion for this illness is mentioned in Table 1.

## 2. Case Presentation

Patient is a 62-year-old white male with a known history of chronic hepatitis C, cirrhosis, ascites, and hypothyroidism. Patient presented to the GI/liver clinic complaining of 1 episode bright red blood per rectum (BRBPR), and exacerbating dyspnea associated with nausea and few episodes of nonbloody vomit. Physical exam showed icterus, jaundice, few small spider angiomas on the chest, decrease breath sounds bilateral right more than left, and that he was mildly tachycardic. Abdominal exam revealed midline scar, moderated size ventral hernia, distention, mild diffused tenderness, and dullness to percussion. Rectal exam did not revealed gross blood. Laboratory result: complete blood count 5.2/13.2/37.6/83, basic metabolic panel was normal LFTs 83/217/125/5.2/4.7/7.4, Pt 22.6 INR 1.9 PTT35.4. Chest X-ray showed right pleural effusion with right middle and lower lobe collapse, and CT scan showed a cirrhotic liver, abdominal varices, and moderated ascites collection around ventral hernia. 

Esophagogastroduodenoscopy showed no esophageal varices. Calculated A-a gradient was 49.5 mmHg; pulmonary function test showed moderated restrictive ventilatory impairment with severe reduction in diffusion capacity. Two-dimensional echocardiography showed a patent foramen ovale (PFO) with predominant left-to-right shunt.

## 3. Discussion

The term “hepatopulmonary syndrome” was first introduced to the academic community in 1977 after compelling findings on autopsy were associated with clinical symptoms [1, 3–5]. The unique pathological finding associated with this disease is an absolute increase of pulmonary precapillary and capillary vessels (15–100 um in diameter at rest) dilation which are visualized by means of injection at autopsy [6]. A few pleural and pulmonary arteriovenous communications (shunts) and portopulmonary venous anastomosis are common findings as well [6]. 

The only consistent pulmonary-function test result in patients with HPS is a decrease in single-breath diffusion capacity. However, this finding is not specific, and may not normalize after transplantation indicating underlying structural remodeling [7]. Researchers have tried to target the theorized pathophysiology behind this condition. Thus, nitric oxide inhibitors, methylene blue (inhibitor of guanylate cyclase and cyclic guanosine monophosphate), or nitric oxide synthase inhibition have shown transient improvements in oxygenation at best [8]. Data from several uncontrolled trials indicate failure with almitrine, antibiotics, beta blockers, COX inhibitors, garlic preparation, systemic glucocorticoids and cyclophosphamide, and somatostatin analogues [9]. Long-term oxygen therapy remains the most frequent recommendation [8]. Experimental studies in which development of HPS was prevented used pentoxifylline, an inhibitor of the production of TNF *α*, suggesting another possible mediator of this condition [10–12].

The only curative therapy for HPS is liver transplant, and in a majority of the cases, within a couple of months, the pathology previously described completely returns to the previous state [12–14]. However, HPS can be quite fatal if no liver transplant is available. In one retrospective study of about 22 patients with HPS, data analysis revealed almost 41% in 2.5-year mortality rate in patients that did not undergo liver transplant. One study reported a 5-year survival of 76% in patient with HPS after successful transplantation. HPS patients with a PO2 less than 60 mmHg, as per a new UNOS policy, can be placed higher on the national liver transplant [15, 16]. 

**Figure 1:**
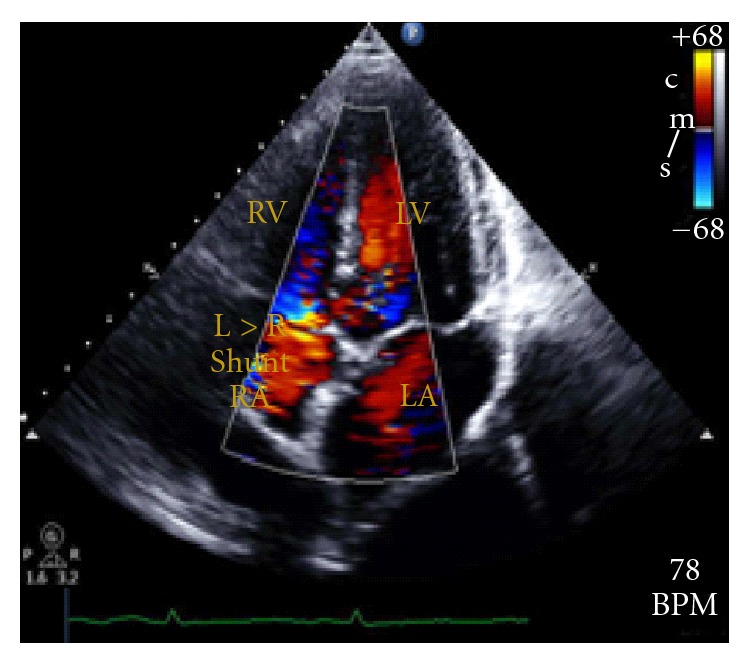
Apical four chamber view showing the presence of a patent foramen ovale with predominant left-to-right shunt.

**Figure 2:**
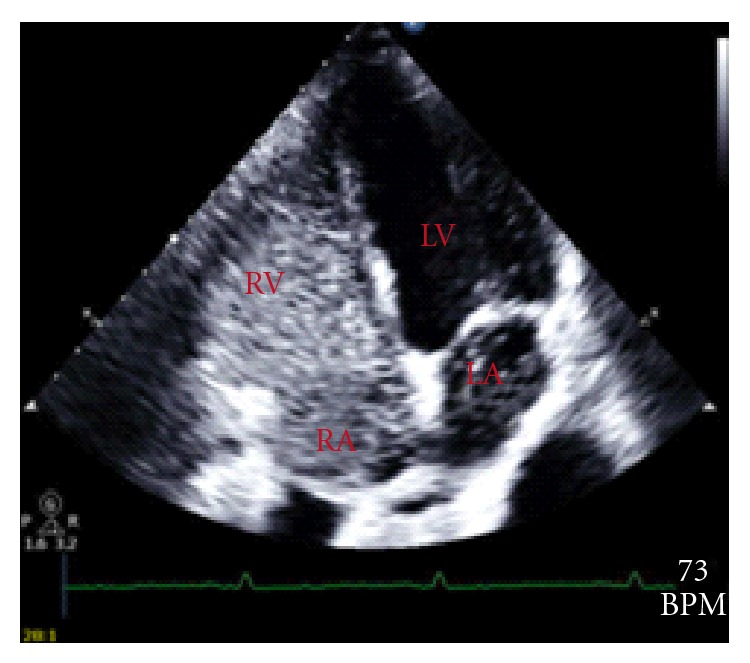
Contrast-enhanced echocardiography showing the appearance of microbubbles in the left atrium 6 cycles after they first appeared in the right.

 During a contrast-enhanced transthoracic echocardiography, contrast is injected peripherally, and under normal circumstances only the right atrium is opacify by this substance. Presence of small amount of contrast material in the left atrium two cardiac cycles after it first appeared in the right atrium suggests the presence of a PFO. However, if this material arrives to the left atrium after 4–8 cardiac cycles prove the presence of dilate pulmonary microvasculature confirming the diagnosis of HPS. In our patient Contrast-enhanced echocardiography showed the presence of a PFO (Figure 1) and the arrival of contrast material to the right atrium 6 cardiac cycles after it was first seen in the left atrium (Figure 2). Existence of this PFO poses a diagnostic challenge since structural heart diseases must be excluded before HPS can be diagnose with certainty [2, 15–17].

According to Aller et al. transesophageal echocardiography is more sensitive for the diagnosis of HPS since it allows visualization of pulmonary vascular bed, and grading of pulmonary vasodilatation [17]. However, severe exertional hypoxemia in our patient, which is one of requirements for HPS diagnosis, cannot be solely attribute to HPS since intermittent right-left shunt secondary to increase intrathoracic pressure (exertion and other physical maneuvers) can be responsible for this symptom [2]. Thus, definitive diagnosis of HPS in this case is hindered by the presence of structural heart disease. 

## Figures and Tables

**Table 1:** Diagnostic criteria for hepatopulmonary syndrome.

Variable	Criterion
Oxygenation defect	Partial pressure of oxygen < 80 mmHg or alveolar-arterial oxygen gradient ≥ 15 while breathing room air
Pulmonary vascular dilation	Positive findings on contrast-enhanced echocardiography or abnormal uptake in the brain (>6%) with radioactive lung-perfusion scanning
Liver disease	Portal hypertension (most common) with or without cirrhosis
Degree of severity	
Mild	A-a∗ oxygen gradient ≥ 15, partial pressure of oxygen ≥ 80 mmHg
Moderate	A-a oxygen gradient ≥ 15, partial pressure of oxygen ≥ 60 mmHg to <80
Severe	A-a oxygen gradient ≥ 15, partial pressure of oxygen ≥ 50 mmHg to <60
Very severe	A-a oxygen gradient ≥ 15, partial pressure of oxygen < 50 mmHg

∗A-a: Alveolar-arterial.

Criterion: [3].

## References

[1] Kennedy T. C., Knudson R. J. (1977). Exercise-aggravated hypoxemia and orthodeoxia in cirrhosis. *Chest*.

[2] Van Gaal W. J., Joseph M., Jones E., Matalanis G., Horrigan M. (2005). Platypnea-orthodeoxia associated with a fenestrated atrial septal aneurysm: case report. *Cardiovascular Ultrasound*.

[3] Hoffbauer F. W., Rydell R. (1956). Multiple pulmonary arteriovenous fistulas in juvenile cirrhosis. *The American Journal of Medicine*.

[4] Fluckiger M. (1884). Vorkommen von trommelschlägelförmigen Fingerendphalangen ohne chronische Veränderungen an den Lungen oder am Herzen. *Wien Med Wochenschr*.

[5] Berthelot P., Walker J. G., Sherlock S., Reid L. (1966). Arterial changes in the lungs in cirrhosis of the liver—lung spider nevi. *The New England Journal of Medicine*.

[6] Rodriguez-Roisin R., Krowka M. J., Herve P., Fallon M. B. (2004). Pulmonary-hepatic vascular disorders (PHD). *European Respiratory Journal*.

[7] Hourani J. M., Bellamy P. E., Tashkin D. P., Batra P., Simmons M. S. (1991). Pulmonary dysfunction in advanced liver disease: frequent occurrence of an abnormal diffusing capacity. *American Journal of Medicine*.

[8] Rodríguez-Roisin R., Krowka M. J. (2008). Hepatopulmonary syndrome—a liver-induced lung vascular disorder. *The New England Journal of Medicine*.

[9] Sztrymf B., Rabiller A., Nunes H. (2004). Prevention of hepatopulmonary syndrome and hyperdynamic state by pentoxifylline in cirrhotic rats. *European Respiratory Journal*.

[10] Zhang J., Ling Y., Tang L. (2007). Pentoxifylline attenuation of experimental hepatopulmonary syndrome. *Journal of Applied Physiology*.

[11] Rodriguez-Roisin R., Krowka M. J. (1994). Is severe arterial hypoxaemia due to hepatic disease an indication for liver transplantation? A new therapeutic approach. *European Respiratory Journal*.

[12] Krowka M. J., Dickson E. R., Cortese D. A. (1993). Hepatopulmonary syndrome: clinical observations and lack of therapeutic response to somatostatin analogue. *Chest*.

[13] Swanson K. L., Wiesner R. H., Krowka M. J. (2005). Natural history of hepatopulmonary syndrome: Impact of liver transplantation. *Hepatology*.

[14] Fallon M. B., Mulligan D. C., Gish R. G., Krowka M. J. (2006). Model for End-Stage Liver Disease (MELD) exception for hepatopulmonary syndrome. *Liver Transplantation*.

[15] Krowka M. J., Tajik A. J., Dickson E. R., Wiesner R. H., Cortese D. A. (1990). Intrapulmonary vascular dilatations (IPVD) in liver transplant candidates. Screening by two-dimensional contrast-enhanced echocardiography. *Chest*.

[16] Abrams G. A., Jaffe C. C., Hoffer P. B., Binder H. J., Fallon M. B. (1995). Diagnostic utility of contrast echocardiography and lung perfusion scan in patients with hepatopulmonary syndrome. *Gastroenterology*.

[17] Aller R., Moya J. L., Moreira V. (1999). Diagnosis of hepatopulmonary syndrome with contrast transesophageal echocardiography: advantages over contrast transthoracic echocardiography. *Digestive Diseases and Sciences*.

